# New Eye Drop Formulation Based on Desonide and Xanthan Gum in Dry Eye Disease: Nonclinical Studies

**DOI:** 10.3390/pharmaceutics17020235

**Published:** 2025-02-12

**Authors:** Santa Viola, Luca Rosario La Rosa, Giuseppe De Pasquale, Manuela Santonocito, Donato Spina, Ilenia Abbate, Francesco Giuliano, Maria Cristina Curatolo, Maria Grazia Mazzone, Cristina Zappulla

**Affiliations:** 1 Innovation and Medical Science, SIFI S.p.A., 95025 Aci Sant’Antonio, Italy; 2 Business Development and Open Innovation, SIFI S.p.A., 95025 Aci Sant’Antonio, Italy

**Keywords:** xanthan gum, desonide sodium phosphate, ocular surface, dry eye

## Abstract

**Background/Objectives:** A novel ophthalmic formulation, XanterDES, containing 0.2% xanthan gum and 0.025% desonide sodium phosphate (DES), was developed to alleviate ocular surface discomfort and irritation. This study aimed to evaluate its pharmacodynamic properties and to characterize its rheological behavior and mucoadhesive characteristics, compared to another formulation containing 0.2% hyaluronic acid and 0.001% hydrocortisone (HYD). **Methods:** A rabbit (New Zealand White) model of LPS-induced uveitis was used to test different concentrations of DES on ocular markers of inflammation. The efficacy of XanterDES and HYD on induced dry eye was evaluated by assessing tear volume and corneal damage in C57BL/6 mice exposed to a controlled environmental chamber. The rheological and mucoadhesive properties of XanterDES and HYD were assessed using a HAAKE RheoStress RS600 rheometer and a TA-XT2 texture analyzer, respectively. **Results:** In the uveitis model, unlike DES 0.25%, a low concentration of 0.025% DES showed a significant inhibitory activity localized to the eye surface and effectively reduced corneal edema. In the dry eye model, XanterDES demonstrated superior efficacy compared to HYD, effectively preventing both tear volume reduction and corneal damage. XanterDES also demonstrated pseudoplastic and enhanced mucoadhesive properties compared to HYD. **Conclusions:** The ancillary anti-inflammatory effects of a low dose of DES combined with the biophysical properties of xanthan gum are supportive of a favorable therapeutic profile, promoting the maintenance or restoration of ocular surface homeostasis while minimizing the risk of adverse effects typically associated with standard-dose corticosteroids. The comparison with another low-dose corticosteroid highlights the superiority of XanterDES in pharmacodynamic and biophysical performance.

## 1. Introduction

Dry eye disease (DED) is a common ocular disorder affecting tens of millions of people. The incidence and prevalence of this ocular disorder in Asia is higher than in Europe and North America, suggesting that cultural or racial factors are involved in disease etiology [1,2]. The prevalence of DED is expected to rise with increasing life expectancy and the growing number of potential risk factors, including environmental factors such as pollution and low humidity, both of which are directly related to climate and lifestyle changes. Additional risk factors include the use of systemic and topical medications, as well as ocular surgeries [3,4,5]. In particular, DED related to prolonged screen use and digital eye strain is becoming increasingly prevalent in both work and leisure activities [3,4,5] even in adolescence and childhood [6].

DED is a multifactorial disease that affects tears and the ocular surface. Key events in the pathogenesis of DED include tear film instability, tear film hyperosmolarity, ocular surface damage, ocular surface inflammation, and visual disturbance [7,8].

The primary goal of DED management is to restore the natural homeostasis of the ocular surface, which can be achieved by interrupting the vicious cycle of pathophysiology [1,9,10]. Treatment typically involves the widespread use of artificial tears containing polymers (e.g., sodium hyaluronate, xanthan gum, methylcellulose, and polyvinyl alcohol), sometimes adjuvated by active principles such as corticosteroids (e.g., fluorometholone, hydrocortisone, prednisolone, and dexamethasone), tetracyclines, and immunosuppressants (e.g., cyclosporine) While tear substitutes are the mainstay of treatment for restoring the ocular surface, moderate-to-severe DED often requires other medications that directly address the underlying pathophysiology of the disease [10,11,12,13,14].

The 2017 Tear Film and Ocular Surface Society Dry Eye Workshop (TFOS DEWS) II report recommends the early initiation of anti-inflammatory therapy with topical corticosteroids for managing DED [8]. At the same time, the Asia Dry Eye Society proposes a very important role of a “normal precorneal tear film” in DED, thus developing a new therapeutic strategy called “tear film oriented therapy” (TFOT). According to the latter, producing a healthy stable tear film is the primary treatment target, although inflammation is not its central concept, and anti-inflammatory drugs are frequently used in the care of dry eye patients [15]. Topical corticosteroids have consistently demonstrated effectiveness in breaking the cycle of inflammation in DED, providing clinical efficacy with short-term use [1,10,16]. Nevertheless, differences regarding the incidence of side effects and evidence of efficacy in dry eye have been reported for the various available topical corticosteroids due to their potency and propensity to penetrate into the anterior chamber [1,17]. Generally, this pharmacological class should be used for a limited duration because of potential adverse events (AEs), such as ocular hypertension, cataracts, and opportunistic infections [1,10]. Nonetheless, corticosteroids like fluorometholone, loteprednol, desonide, and hydrocortisone may be considered safer options, as they have a lower likelihood of causing these side effects [1,10,13].

On this premise, we have developed XanterDES, a novel ophthalmic formulation designed for the management of ocular surface discomfort. The product contains 0.2% xanthan gum (XNT) and 0.025% desonide sodium phosphate (DES), a low concentration of a corticosteroid selected to minimize potential ocular and systemic side effects and localize the therapeutic action to the eye surface.

XNT is a high-molecular-weight anionic polysaccharide capable of forming viscoelastic gels. It is known for its lubricating, antioxidant, and immunomodulatory properties [12,18,19]. XNT also acts as a scaffold in re-epithelialization processes [12,20]. Furthermore, to perform its antioxidant and mucoadhesive properties, the polymer ameliorated the conjunctival epithelium of mild-to-moderate dry eye patients [21]. The interaction of XNT with the mucinous layer of the tear film is supported by its physicochemical properties and structural characteristics [22].

In addition to the presence of XNT, the new eye drop formulation contains a low concentration of 0.025% DES with ancillary action. Desonide is a medium-potency corticosteroid originally developed for dermatologic use. The desonide sodium phosphate ophthalmic ointment and solution at a concentration of 0.25% has been available for many years in Italy. DES is a nonfluorinated corticosteroid with a nonsymmetric 16, 17-acetyl substitution, which is believed to result in low ocular penetration and absorption. This molecule has been successfully used to treat allergic conjunctivitis without significantly increasing intraocular pressure (IOP) [17,23,24].

More recently, an observational clinical study demonstrated the efficacy of this new eye drop formulation containing 0.2% XNT and 0.025% DES in patients with mild-to-moderate DED, showing a good safety and performance profile throughout the one-month treatment course [14].

On this ground, the purpose of the present study was threefold: first, to investigate the anti-inflammatory efficacy of a low 0.025% DES concentration in a rabbit model of uveitis compared to 0.25% and 0.0025% DES; second, to assess the efficacy of XanterDES in a murine model of dry eye compared to another eye drop formulation containing 0.2% hyaluronic acid and 0.001% hydrocortisone (hereafter referred to as HYD); and third, to evaluate the rheological profile and mucoadhesive properties of XanterDES compared to HYD.

## 2. Materials and Methods

### 2.1. Materials

DES was purchased from Farmabios (Pavia, Italy). XanterDES was from SIFI S.p.A. (Aci S. Antonio, Catania, Italy). Bacterial lipopolysaccharide (LPS) from *E. coli* (serotype 055: B5, L2637), fluorescein sodium salt, scopolamine hydrobromide, and dried mucin from porcine stomach type II were from Sigma Aldrich (Milan, Italy). Sodium chloride, sodium hydrogen carbonate, calcium chloride bihydrated, and potassium chloride were purchased from Merck (Milan, Italy). Zone Quick lacrimedics was from FCI Ophthalmics Inc (Pembroke, MA, USA). The eye drop formulation for HYD was from Alfa Intes (Naples, Italy). Trypan blue was from Life Technologies (Milan, Italy). The prostaglandin E2 (PGE_2_) enzyme immunoassay (EIA) kit was from Cayman Chemical Company (Ann Arbor, MI, USA). Zoletil 50 + 50 mg/mL was from Virbac (Milan, Italy). Domitor 1 mg/mL was from Orion Pharma s.r.l. (Milan, Italy). Tanax was purchased from MSD Animal Health S.r.l. (Milan, Italy). Calciparine 5000/0.2 mL was from Italfarmaco S.p.A. (Milan, Italy).

### 2.2. Rabbit Model of Endotoxin-Induced Uveitis

Forty male New Zealand albino rabbits within the required weight range of 1.8–2.2 kg and with an approximate age of 7–11 weeks were obtained from Harlan (Milan, Italy). Rabbits, fed with standard laboratory food and with water ad libitum, were allocated in the animal rooms of SIFI Laboratories, with one animal per cage. The animal rooms were maintained at standard conditions of humidity (40–60% RH), temperature (15–21 °C), and artificial lighting (12 h light and 12 h dark for each day). All the animals were treated according to the Association for Research in Vision and Ophthalmology (ARVO) Statement for the Use of Animals in Ophthalmic and Vision Research and the Directive 86/609/CEE of the Council of the European Communities. The protocols were approved by the Italian Ministry of Health (authorization no. 50/2007-B of 21 May 2007).

Following anesthesia, by means of intramuscular administration of Domitor and Zoletil, a Hamilton syringe fitted with a 30-gauge needle was used to deliver a 10 μL volume containing 100 ng of LPS intravitreally into the right eye of each rabbit [25]. The injection was performed through the pars plana of the eye by directing the needle posterior towards the antero–posterior axis. Animals were treated after the LPS injection (T = 0) and then 3, 6, and 9 h later. The left eye of all animals remained untreated.

For treatments, animals were divided into four groups and topically treated (55 μL drop size) with DES 0.25%, DES 0.025%, DES 0.0025%, or saline (VHC).

Corneal thickness was measured via pachymetry prior to the LPS injection (Baseline, T0), 9, and 24 h later. Animals were anesthetized with Domitor and Zoletil, followed by euthanasia via the intravenous injection of Tanax. The aqueous humor from the right eye of each animal was withdrawn. A 1 mL plastic syringe fitted with a 30-gauge needle was used to perforate the cornea peripherally, and the aqueous humor was gently aspirated from the anterior chamber. To avoid the formation of cellular aggregates, aqueous humor samples were supplemented with 5 μL of saline containing 25 U of calciparine. The sample was then stored on ice until further analysis. From each aqueous humor sample, 50 μL were centrifuged to remove cells and tissue debris, and the supernatant was assayed for PGE_2_ content measurements via EIA. The remainder of the sample was used for assessing leukocyte numbers using trypan blue.

### 2.3. Dry Eye Mouse Model

Thirty-two C57BL/6 female mice (within a weight range of 15–18 g) with an approximate age of 8–12 weeks were obtained from Charles Rivers Laboratories Italia S.r.l. (Milan, Italy) and housed in the animal rooms of SIFI S.p.A. The animals were provided with standard pellet food (Mucedola; Milan, Italy) and tap water ad libitum. All procedures involving animals were conducted in accordance with the Italian Legislative Decree 4 March 2014, No. 26. Implementation of Directive 2010/63/EU on the protection of animals used for scientific purposes, and with the ARVO Statement for the Use of Animals in Ophthalmic and Vision Research. The study protocols were approved by the Italian Ministry of Health (authorization no. 580/2018 dated 31 July 2018; protocol no. 22CE8.6.EXT.8 dated 23 February 2022).

Four experimental sets were conducted, each involving eight animals divided into four groups of two mice each. The mice inside the controlled environmental chamber (CEC; modified from Barabino et al., 2005) were divided into three groups: (1) the untreated positive control group (CTRL+, n = 8); (2) treated with XantherDES (n = 8); and (3) treated with HYD (n = 8). Additionally, a negative control group (CTRL−, n = 8) was kept outside the CEC [26] in standard environmental conditions, i.e., 21–23 °C and 50–60% relative humidity (RH).

Mice inside the CEC were subjected for 3 days to low humidity (5 ≤ RH ≤ 15) and constant airflow (≈20 L/min). These conditions were ensured by means of an oxygen generator connected to the CEC. In addition to the environmental conditions, animals inside the CEC received subcutaneous injections of scopolamine (10 µL of a 5 mg/mL solution) three times daily for three days in order to induce (or simulate) both evaporative and aqueous-deficient dry eye.

Seven microliters of the test items were instilled in both eyes three times daily for 3 days; animals outside the CEC, i.e., CTRL−, were left untreated for the entire duration of the study.

Corneal damage was evaluated via fluorescein staining with a slit lamp at baseline and after 3 days of treatment (T3).

For the observation with blue light, a drop (1 µL) of a sterile solution of 1% fluorescein was administered into the inferior conjunctival sac of the mice’s eyes. After administration, the eyelid was kept closed for a few seconds to allow the fluorescein to distribute and adsorb onto the ocular surface; then, each eye was thoroughly rinsed with a sterile 0.9% NaCl solution to remove any unbound fluorescein.

Punctate staining was assessed using the standardized National Eye Institute grading system, with a score from 0 to 3 assigned to each of the five designated corneal regions [27], resulting in a maximum total score of 15.

Tear volume was measured in both eyes at baseline (T0) and after 3 days of treatment (T3) using the cotton thread test. Under a magnifying lamp, the threads were held with forceps and placed in the conjunctival fornix for 1 min. The tear volume, expressed as the length in millimeters of thread wet by tear fluid, was read under a stereo microscope using a micrometric scale and analyzed with the ImageJ software (version 1.52a) [28].

### 2.4. Rheological Properties

Rheological measurements were carried out using a HAAKE RheoStress RS 600 (Thermo Fisher Scientific Inc.; Waltham, MA, USA) rotational rheometer equipped with a cone-plate geometry (60 mm diameter, 1° cone angle). The experimental setup was adapted from Kapadia et al., 2022 [29]. The dynamic viscosity of the samples was recorded as a function of shear rate increasing from 0.01 to 8500 s^−1^ at 25 °C over a period of 900 s. XanterDES and a HYD were tested in duplicate, so the viscosity values (η, mPa∙s) were considered as the mean of two measurements. From each flow curve, the rheological behavior was obtained, and the measurements of viscosity (mPa∙s) at 10 s^−1^ and 8000 s^−1^ were extrapolated.

### 2.5. Mucoadhesion Study

The adhesive properties of XanterDES compared to HYD were evaluated using a TA-XT2 texture analyzer equipped with a 5 kg ± 0.1 g load cell (Stable Micro Systems, Surrey, UK) [30]. The setup included an upper movable ebonite cylinder probe (12.7 mm in diameter) and a lower fixed cylinder (20.0 mm in diameter), each fitted with disks attached via double-sided adhesive tape. The mucin dispersion (4% *w*/*w* mucin from porcine stomach type II) was made in simulated tear fluid (STF) at pH = 7.4. The STF composition was as follows: 6.8 g of sodium chloride, 2.2 g of sodium hydrogen carbonate, 0.08 g of bi-hydrated calcium chloride, and 1.4 g of potassium chloride in 1 L of purified water. The final pH of the STF solution was adjusted to the value of 7.4 with 1N hydrochloric acid. For each formulation, two test systems were compared: mucin–mucin interactions (as the reference) and mucin–formulation interactions. The instrument was configured to bring the upper probe into contact with the lower one, measuring the force during both penetration and withdrawal by applying a contact force for 120 s. Key settings included a pre-test speed of 1 mm/s, a test speed of 0.5 mm/s, a post-test speed of 0.1 mm/s, and a data acquisition rate of 100 points per second. The probe penetration depth into the formulation was set at 0.1 mm, with a trigger force of 0.008 N and automatic tare mode enabled, and set to return to the starting position. Approximately 0.04 g of mucin dispersion was evenly spread over the probe, which was then brought into contact with 0.20 g of the mucin/formulation mixture at a 1:1 ratio and held for 120 s. To ensure data accuracy and reproducibility, at least six measurements were performed for each condition.

The force required to separate the mucin-coated probe from the formulation mixture was recorded and displayed as a profile of negative force (F) against either time (s) or distance (D, the distance measured in millimeters until complete detachment occurred). Mucoadhesion occurs when the adhesive forces between mucin and formulation polymers exceed repulsive forces, enhancing the mucin–formulation interface. This is quantified by a higher detachment work (Wmucoad) compared to the cohesive work within mucin (Wmuc), indicating stronger interfacial bonding. Detachment work, derived from the force–distance curve, represents the energy needed to disrupt the weakest bonds at the mucin–formulation interface.

### 2.6. Statistical Analysis

All statistical analyses were conducted using the GraphPad Prism 6 software (San Diego, CA, USA). In all statistical tests, the significance threshold was set at *p* < 0.05.

Rabbit model of endotoxin-induced uveitis: significant differences between groups were sought using one-way ANOVA followed by Holm–Sidak post hoc test.

Dry eye mouse model: The scores obtained from the observation under slit lamp were statistically analyzed as non-parametric values using Kruskal–Wallis test plus Dunn’s post hoc test. Measurements obtained with the cotton thread test (parametric values) were analyzed via one-way ANOVA plus Dunnet’s post hoc test.

Mucoadhesion study: statistical analysis was performed via the one-way ANOVA test followed by Tukey’s multiple comparison test.

## 3. Results

### 3.1. Effect of a Low Concentration of DES on Inflammatory Parameters in a Model of Endotoxin-Induced Uveitis

The effect of three different concentrations of DES was investigated in a rabbit LPS-induced uveitis model in order to identify the one that could spare intraocular structures while exerting anti-inflammatory activity on the ocular surface. Eye drops (55 µL) containing DES 0.25%, 0.025%, 0.0025%, or VHC were administered immediately after and then 3, 6, and 9 h following the LPS intravitreal injection. Corneal thickness, leukocytes, and PGE_2_ in the aqueous humor were measured.

**Figure 1:**
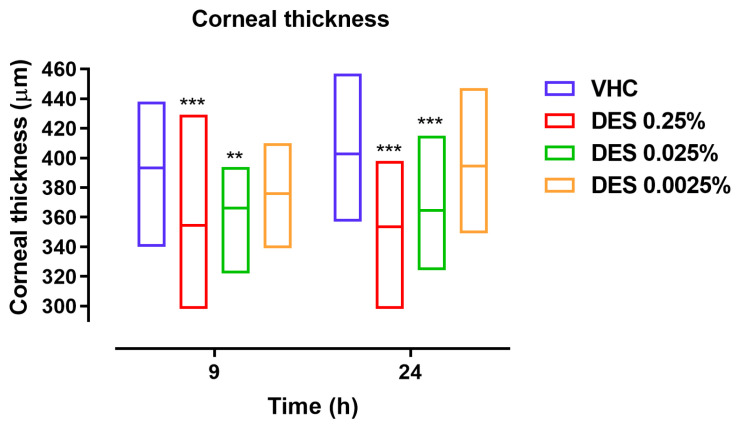
Effect of different concentrations of desonide disodium phosphate (DES) on corneal thickness at 9 and 24 h from the intravitreal injection of the lipopolysaccharide (LPS). Data are graphed as floating bars, spanning from minimum to maximum, and as a line at the mean value of measurements in 10 animals per group. ** *p* ≤ 0.01, *** *p* ≤ 0.001. One-way ANOVA plus Holm–Sidak post hoct test, DES vs. saline (VHC).

Results demonstrated that the intravitreal injection of LPS induced an increase in corneal thickness in VHC-treated animals, reaching 9% and 11% more than baseline values (359 ± 16 μm) at 9 and 24 h from insult, respectively. The corneal edema induced by LPS was significantly inhibited by DES 0.25% and 0.025%, reducing corneal thickness on average by 92% (*** *p* ≤ 0.001 vs. VHC) and 79% (** *p* ≤ 0.01 vs. VHC) at 9 h and by 100% and 83% at 24 h (*** *p* ≤ 0.001 vs. VHC), respectively (Figure 1). In contrast, DES 0.0025% was found to have no statistically significant effect on corneal thickness (Figure 1).

**Figure 2:**
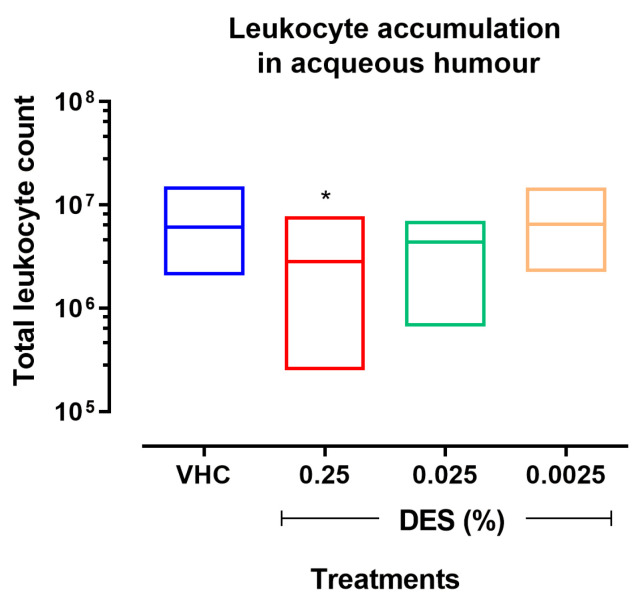
Effect of different concentrations of desonide disodium phosphate (DES) on total leukocyte number in the aqueous humor at 24 h from the intravitreal injection of the lipopolysaccharide (LPS). Data are graphed as floating bars, spanning from minimum to maximum, and as a line at the mean value of measurements in 10 animals per group. * *p* < 0.05, one-way ANOVA plus Holm–Sidak post hoc test, DES vs. saline (VHC).

**Figure 3:**
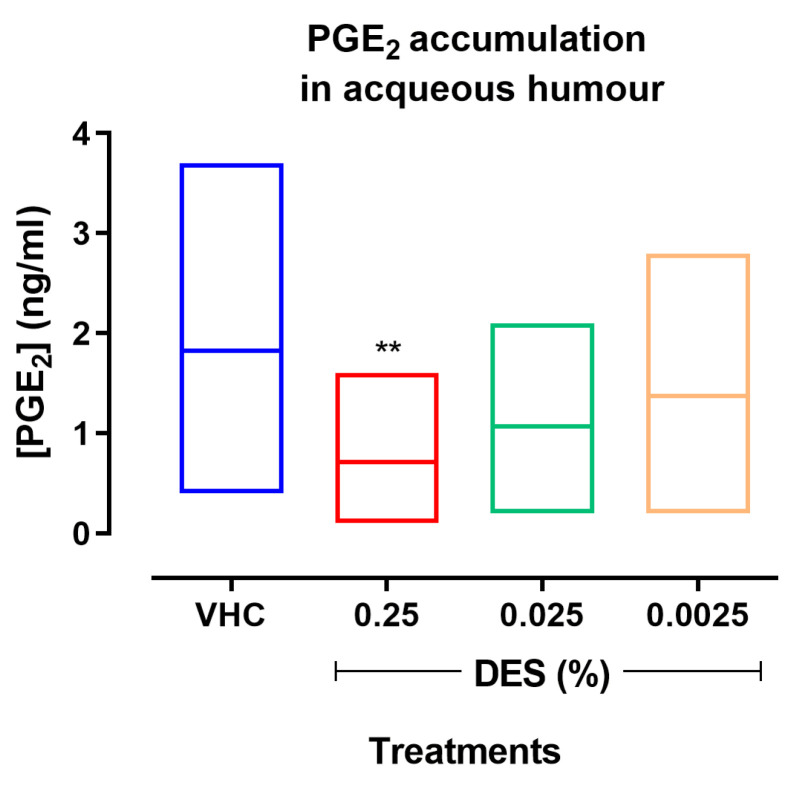
Effect of different concentrations of desonide disodium phosphate (DES) on prostaglandin E2 (PGE_2_) accumulation in the aqueous humor at 24 h from the intravitreal injection of the lipopolysaccharide (LPS). Data are graphed as floating bars, spanning from minimum to maximum, and as a line at the mean value of measurements in 10 animals per group. ** *p* < 0.01, One-way ANOVA plus Holm–Sidak post hoc test, DES vs. saline (VHC).

Similarly, when compared to VHC animals, DES 0.0025% failed to produce any significant effect on the accumulation of leukocytes or PGE_2_ in the aqueous humor (Figure 2 and Figure 3). On average, DES 0.25% and 0.025% inhibited the accumulation of leukocytes by 35% (* *p* ≤ 0.05 vs. VHC) and 16% (Figure 2), as well as the accumulation of PGE_2_ by 45% (** *p* ≤ 0.001 vs. VHC) and 44%, respectively (Figure 3). However, only DES 0.25% did produce effects regarding the accumulation of leukocytes (* *p* ≤ 0.05 vs. VHC) and PGE_2_ (** *p* ≤ 0.01 vs. VHC), also attaining statistical significance (Figure 2 and Figure 3).

### 3.2. Efficacy of XanterDES in a Mouse Model of Dry Eye Compared to Another Medical Device

An evaporative and aqueous-deficient model of DED in mice was used to evaluate the effect of XanterDES compared to another marketed medical device, i.e., HYD. C57BL/6 mice were subjected for 3 days to the CEC with low humidity, constant airflow, and the scopolamine s.c. injection. In parallel, a group of CTRL- was kept in standard environmental conditions outside the CEC. Animals were divided into the following groups: (1) un-treated CTRL+; (2) treated with XanterDES; (3) treated with HYD; and (4) un-treated CTRL-. Test items were instilled in both eyes three times daily for 3 days. Corneal damage (slit lamp) and tear volume (cotton thread) were evaluated at baseline and after 3 days of treatment (T3).

**Figure 4:**
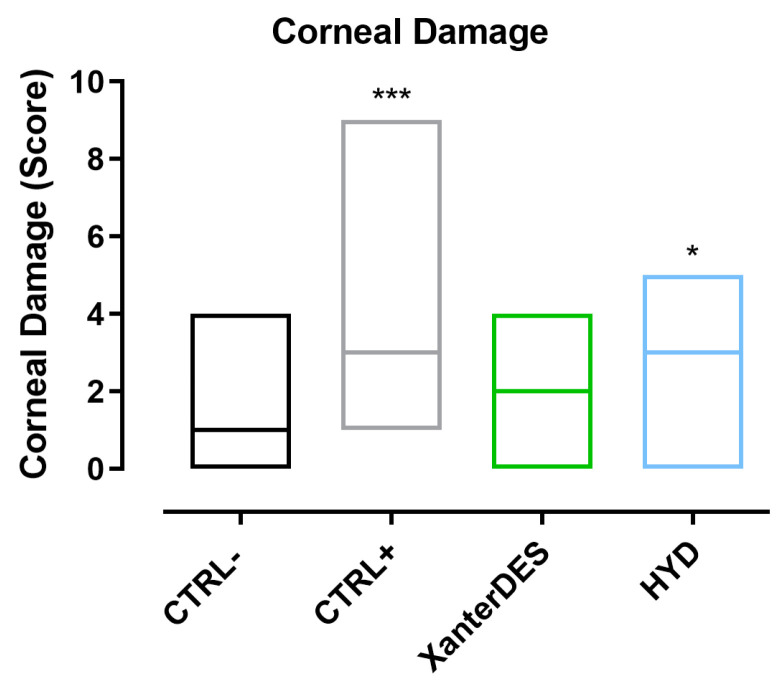
Corneal damage measured via fluorescein staining at 3 days of treatment in control negative (CTRL−), control positive (CTRL+), XanterDES-treated, and eye drop formulation based on 0.2% hyaluronic acid and 0.001% hydrocortisone-treated (HYD) mice. The scores assigned to each group at day 3 of treatment were graphed as floating bars spanning from maximum to minimum with a line at the median (n = 8 per group). Statistical analyses were performed using Kruskal–Wallis plus Dunn post hoc test. * *p* ≤ 0.05, *** *p* ≤ 0.001; vs. CTRL−.

Data gathered on corneal damage demonstrated that CTRL+ mice exhibited significant corneal damage after 3 days compared to CTRL− mice (*** *p* ≤ 0.001, Figure 4). Similarly, HYD did not show a significant protective effect, as corneal damage was found to be increased and statistically different from CTRL− (* *p* ≤ 0.05, Figure 4). Importantly, XanterDES-treated mice did not differ from CTRL−, demonstrating that XanterDES prevented damage induced by exposure to the CEC (Figure 4).

**Figure 5:**
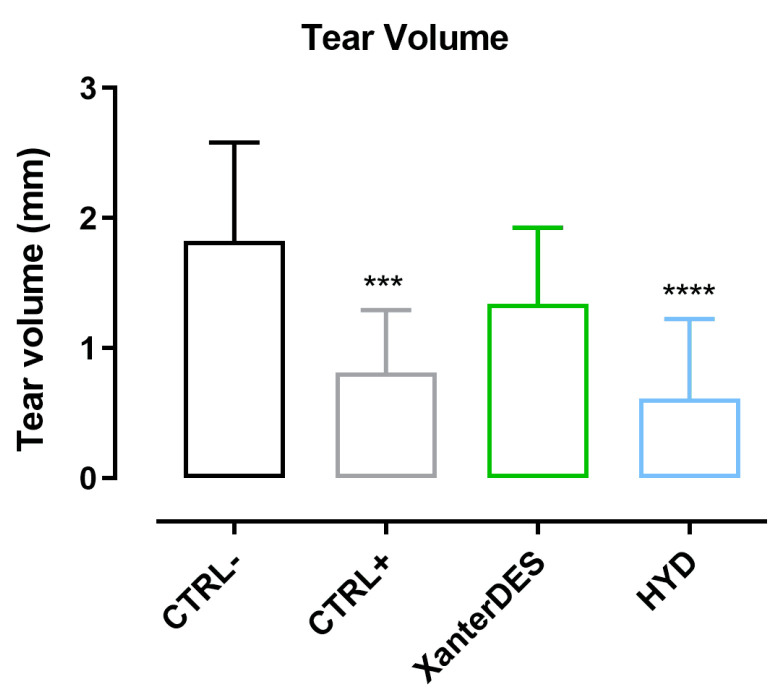
Tear volume using a cotton thread at 3 days of treatment in control negative (CTRL−), control positive (CTRL+), XanterDES-treated, and eye drop formulation based on 0.2% hyaluronic acid and 0.001% hydrocortisone-treated (HYD) mice. Tear volumes in millimeters were graphed as histograms (n = 8 per group). Statistical analyses were performed using one-way ANOVA followed by Dunnett post hoc-test. *** *p* ≤ 0.001, **** *p* ≤ 0.0001; vs. CTRL−.

Moreover, the tear volume of CTRL+ mice was found to be significantly lower compared to CTRL− (*** *p* < 0.001, Figure 5). Consistent with observations on corneal damage, HYD-treated mice showed a significant reduction in tear volume compared to CTRL− mice (**** *p* ≤ 0.0001, Figure 5). Importantly, XanterDES maintained tear volume at levels comparable to CTRL− mice, indicating its protective effect (Figure 5).

### 3.3. Rheological Properties of XanterDES Compared to Another Medical Device

**Figure 6:**
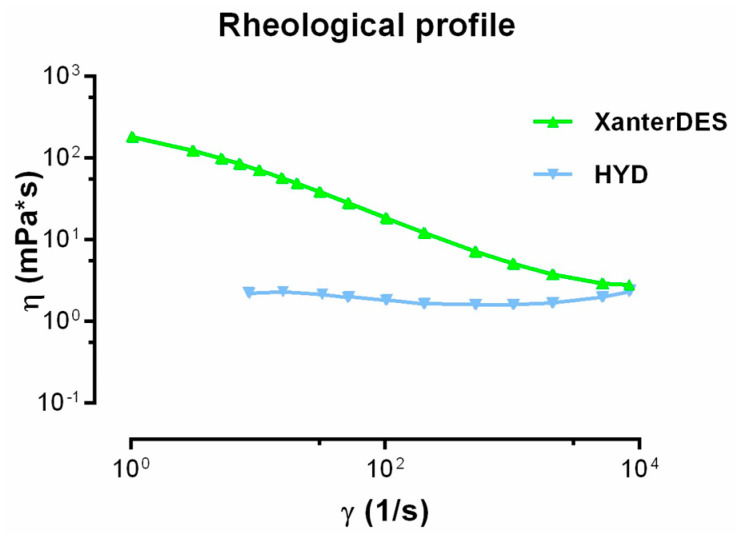
Rheological profile of Xanterdes and of an eye drop formulation based on 0.2% hyaluronic acid and 0.001% hydrocortisone (HYD) evaluated based on a shear rate ranging from 1 to 8500 s^−1^.

XanterDES exhibited a pseudoplastic behavior. Its viscosity varied from 70 mPa·s (at 10 s^−1^) to 2.8 mPa·s (at 8000 s^−1^) (Figure 6). In contrast, HYD showed a constant viscosity under shear stress, indicating a Newtonian behavior with viscosity values of 2.4 and 2.3 mPa·s at 10 s^−1^ and 8000 s^−1^, respectively (Figure 6).

### 3.4. Mucoadhesion Study on XanterDES Compared to Another Medical Device

**Figure 7:**
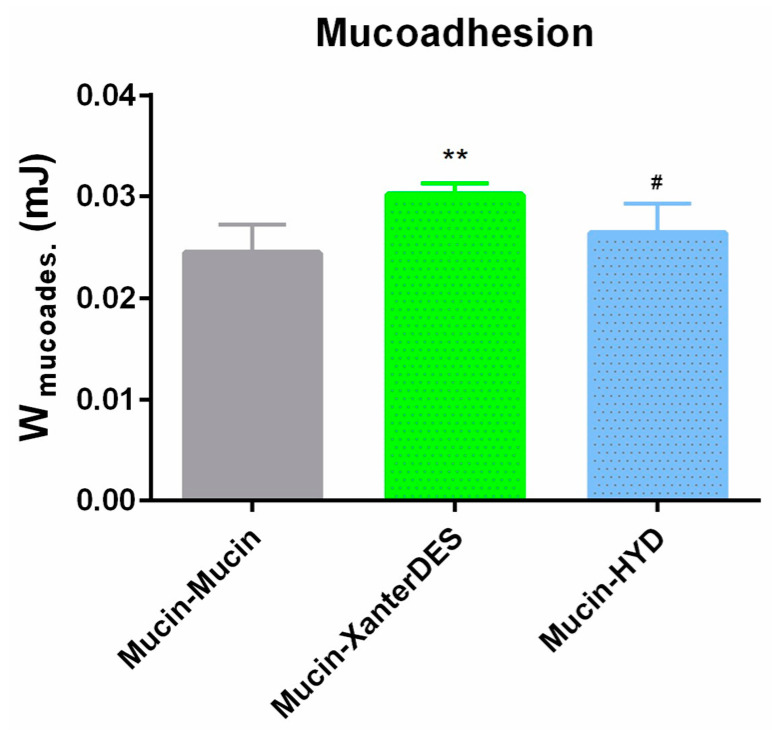
Mucoadhesion profile of Mucin–Mucin, Mucin–XanterDES, and Mucin–HYD. ** *p* ≤ 0.01 vs. Mucin–Mucin. # *p* ≤ 0.05 vs. Mucin–XanterDES. One-way ANOVA plus Tukey’s multiple comparison test.

For XanterDES, Wmucoad was higher than Wmuc (** *p* ≤ 0.01), indicating a significant interaction between the formulation and the mucin layer (Wmucoad > Wmuc), as shown in Figure 7. Specifically, the work of adhesion (Wmucoad) was 30.1.6 ± 1.10 × 10^−3^, in contrast to Wmuc, which measured at 24.4 ± 2.76 × 10^−3^. In the case of HYD, Wmucoad (26.4 ± 2.88 × 10^−3^) was not statistically different than Wmuc, suggesting some interactions between the formulation and mucin, though less pronounced than that of XanterDES (Figure 7). Moreover, statistical analysis confirmed a significant difference (# *p* ≤ 0.05) in Wmucoad for XanterDES when compared to Wmucoad for HYD, highlighting the superior mucoadhesive properties of XanterDES (Figure 7).

## 4. Discussion

DED is a significant global public health concern, and no universally safe and effective treatment is currently available [31]. Despite substantial advances in our understanding of DED over the past two decades, many aspects of the disease, including its epidemiology, biochemistry, physiology, diagnosis, and treatment, remain incompletely understood [32].

The ocular surface epithelium and tear film contain various cells, including immune cells such as dendritic cells, macrophages, mast cells, and lymphocytes, as well as fibroblasts. The latter, along with cytokines and other immune mediators, protect against pathogens and environmental threats. DED can develop when this homeostasis is disrupted by factors such as aqueous tear deficiency, tear film hyperosmolarity, and inflammation of the ocular surface microenvironment [2]. Additional contributors to ocular surface inflammation may include microtrauma, aging, infections, irritants like UV light, and systemic inflammatory diseases, including autoimmune and alloimmune disorders, neuroinflammation, and sterile inflammation. In the early stages of inflammatory DED, these factors can trigger the production of inflammatory cytokines and subsequent immune responses [2].

Recent research has highlighted the role of “para-inflammation” in modulating the progression of DED [13,33,34].

Para-inflammation is a protective phenomenon that exists between homeostasis and inflammation. It has intermediate characteristics within basal and inflammatory states that can become deleterious if stimuli persist. This adaptive response does not require overt tissue injury or infection but is elicited by tissue malfunction and is switched off as soon as homeostasis is restored. However, if stimuli persist over time, para-inflammation can shift from a beneficial and protective response to a detrimental and damaging process, inducing a continuous inflammatory state, which has been associated with different chronic diseases. In patients with DED, this condition plays a pivotal role, as the ocular surface can enter the vicious cycle of the disease but also has the potential to return to a homeostatic state [33].

Thus, actively managing para-inflammation in the early stages of DED is crucial to prevent the exacerbation of ocular surface inflammation and the chronic progression of the disease. While several treatments, such as topical corticosteroids, cyclosporine, and integrin antagonists, are available to manage the inflammatory process, there remains a need for better-tolerated and more effective therapies [35].

A viable approach for the effective and safer use of corticosteroids in mild/moderate ailments of the ocular surface may be represented by eye drops containing low concentrations of this class of molecules.

Recently, the effect of a treatment with 0.2% hyaluronic acid in combination with 0.001% low-dose hydrocortisone in controlling moderate DED has been demonstrated [13,36]. More recently, an observational clinical study demonstrated the efficacy of the new eye drop formulation containing 0.2% XNT and 0.025% DES in patients with mild-to-moderate DED, showing a good performance and safety profile after one month of treatment [14].

**Figure 8:**
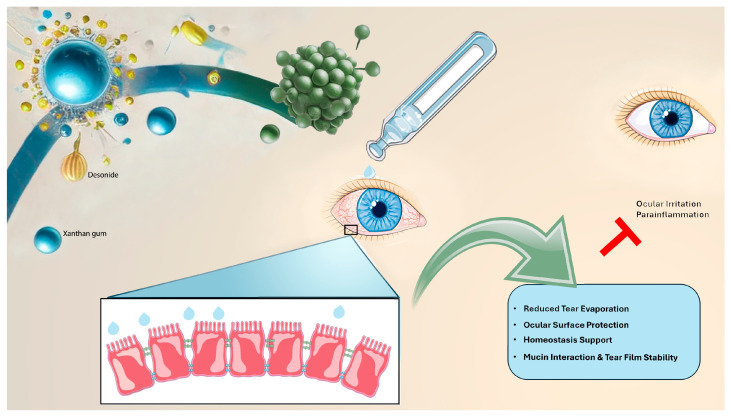
Xanthan gum (blue) and desonide (yellow) combination in dry eye conditions. XanterDES formulation (green) restores ocular surface homeostasis by reinforcing tear film stability, supporting mucin distribution, and minimizing tear evaporation. Additionally, it inhibits para-inflammation, thus preventing ocular irritation and the progression of ocular surface damage.

The combination of XNT and DES offers a multifaceted therapeutic approach to the ocular surface protection. This formulation provides a biophysical support to the tear film, addressing multiple pathological aspects of DED (Figure 8). The polymeric component reinforces the mucin layer distribution by enhancing tear film stability and reducing fluid loss. By forming a viscoelastic network on the ocular surface, XNT decreases the evaporation rate of the tear film, ensuring prolonged hydration. This is particularly crucial in conditions where the tear film is unstable or deficient, preventing excessive moisture loss and improving overall tear retention. DES plays a crucial role in the management of para-inflammation by preventing ocular surface inflammation. Therefore, XanterDES, due to the presence of both components, plays a crucial role in protecting the ocular surface from progressive dysfunction, maintaining the tear film integrity, reducing the risk of chronic dry eye progression and related complications (Figure 8).

On these premises, this study investigates the anti-inflammatory efficacy of different concentrations of DES in a rabbit model of uveitis and the efficacy of XanterDES in a murine model of dry eye compared to another eye drop formulation based on 0.2% hyaluronic acid and 0.001% hydrocortisone, i.e., HYD, and it evaluates the rheological profile of both formulations.

Firstly, the effect of three different concentrations of DES (i.e., 0.0025%, 0.025%, and 0.25%) was investigated in a rabbit model of LPS-induced uveitis to identify the one that could spare intraocular structures while exerting anti-inflammatory activity on the ocular surface. Endotoxin-induced uveitis is an efficient experimental model to study the pathological mechanisms associated with the disease and to evaluate the pharmacological efficacy of potential new drugs [25,37]. Results in the uveitis model demonstrated that DES, a medium-potency corticosteroid, when administered at concentrations matching those used in clinical practice, i.e., 0.25% [17], effectively inhibited inflammation in the animal model of uveitis. Most importantly, DES 0.025%, a concentration 10 times lower than that used in clinical practice, retained a notable degree of activity that was statistically significant only on corneal edema and not on anterior chamber inflammatory markers (i.e., leukocytes and prostaglandin E2), providing grounds in support of a favorable safety profile. Based on the results obtained from testing different concentrations of desonide sodium phosphate, the 0.025% concentration was selected as the target concentration for XanterDES formulation. Thus, the 0.025% concentration was identified as the most appropriate for ensuring both efficacy and tolerability in XanterDES. Preliminary studies on the biocompatibility of XanterDES on human corneal epithelial (HCE) cells demonstrated the cytocompatibility of the formulation when tested undiluted [38].

The efficacy of XanterDES compared to HYD was investigated in a murine model of dry eye. The model used in this study mimics both the hyposecretive and aqueous-deficient forms of DED, inducing a decrease in tear volume and an increase in ocular surface damage in C57BL/6 mice [26].

The effects observed in dry eye animals following treatment with XanterDES suggest that this medical device, due to the presence of XNT and DES, limits the reduction in the tear film and assists to inhibits the onset of inflammatory phenomena associated with environmental insults, due to the combined action of its components.

Different artificial tear products containing viscosifying macromolecules (e.g., sodium hyaluronate, xanthan gum, and methylcellulose) are commercially available for the management of DED [11]. Among these, xanthan gum, due to its high molecular weight and water-retention properties, forms a viscous layer on the eye surface, thereby reducing mechanical friction during eyelid movement and preventing irritation and discomfort, especially in dry eye syndrome. Xanthan gum creates a protective bioadhesive film on the ocular surface, mimicking the natural mucous layer. This film acts as a barrier against desiccation (dryness). It enhances tear film stability by interacting with the aqueous and mucous layers, preventing tear film breakup and evaporation. Its bioadhesive nature ensures prolonged retention on the ocular surface, increasing the therapeutic efficacy and protection [12,39,40,41].

Notably, XanterDES contains a 0.2% XNT polymer. XNT is a branched polymer with important moieties such as pyruvic esters. It has a high intrinsic viscosity and a pronounced pseudoplastic flow at relatively low concentrations due to its functional properties to create a network structure via hydrogen bonding particularly in aqueous systems [40,42]. The rheological profile and mucoadhesive properties of XanterDES were evaluated in comparison to another medical device, i.e., HYD. XanterDES exhibited viscoelastic behavior with a characteristic rheological curve attributed to the presence of XNT. This is characterized by increased low-shear viscosity to improve retention time and reduced high-shear viscosity to minimize blur and stickiness during blinking. On the contrary, the viscosity of HYD remains low and constant with increasing applied force, i.e., typical behavior of Newtonian fluids. In mucoadhesion studies, XanterDES demonstrated significantly improved adhesive properties compared to both the mucin–mucin control and HYD. This increased mucoadhesion likely contributes to prolonged ocular surface contact, which may result in improved therapeutic efficacy. These results highlight the critical role of mucoadhesion in ocular formulations, demonstrating a superior interaction with mucins, potentially enhancing drug delivery and patient outcomes.

The findings presented in this study, when considered alongside previously published data on dry eye patients [14], support the hypothesis that a low concentration of 0.025% DES has the potential to spare intraocular structures while still providing an anti-inflammatory effect on the ocular surface. Further study can be performed to substantiate this hypothesis.

## 5. Conclusions

These data collectively suggest that XanterDES may offer a viable approach for treating mild-to-moderate DED by combining the peculiarities of a branched, pseudoplastic, and mucoadhesive polymer like XNT with the ancillary anti-inflammatory properties of low-dose DES. The comparison with another low-dose corticosteroid, i.e., HYD, highlights the superiority of XanterDES in pharmacodynamic and biophysical performance.

In conclusion, due to the presence of 0.2% xanthan gum and 0.025% DES, XanterDES is able to hydrate, lubricate, and contribute to ocular surface homeostasis in DED patients.

## Data Availability

The original contributions presented in this study are included in the article. Further inquiries can be directed to the corresponding author.
